# Untargeted Metabolomics Reveals Acylcarnitines as Major Metabolic Targets of Resveratrol in Breast Cancer Cells

**DOI:** 10.3390/metabo15040250

**Published:** 2025-04-05

**Authors:** Isabella G. Falcone, Blake R. Rushing

**Affiliations:** 1 Department of Nutrition, University of North Carolina at Chapel Hill, Chapel Hill, NC 27599, USA; 2 Nutrition Research Institute, University of North Carolina at Chapel Hill, Kannapolis, NC 28081, USA

**Keywords:** metabolomics, resveratrol, breast cancer, triple-negative, mitochondria

## Abstract

**Background/Objectives:** Millions of new diagnoses of breast cancer are made each year, with many cases having poor prognoses and limited treatment options, particularly for some subtypes such as triple-negative breast cancer. Resveratrol, a naturally occurring polyphenol, has demonstrated many anticancer properties in breast cancer studies. However, the mechanism of action of this compound remains elusive, although prior evidence suggests that this compound may work through altering cancer cell metabolism. Our objective for the current study was to perform untargeted metabolomics analysis on resveratrol-treated breast cancer cells to identify key metabolic targets of this compound. **Methods:** MCF-7 and MDA-MB-231 breast cancer cells were treated with varying doses of resveratrol and extracted for mass spectrometry-based untargeted metabolomics. Data preprocessing and filtering of metabolomics data from MCF-7 samples yielded 4751 peaks, with 312 peaks matched to an in-house standards library and 3459 peaks matched to public databases. **Results:** Pathway analysis in MetaboAnalyst identified significant (*p* < 0.05) metabolic pathways affected by resveratrol treatment, particularly those involving steroid, fatty acid, amino acid, and nucleotide metabolism. Evaluation of standard-matched peaks revealed acylcarnitines as a major target of resveratrol treatment, with long-chain acylcarnitines exhibiting a 2–5-fold increase in MCF-7 cells and a 5–13-fold increase in MDA-MB-231 cells when comparing the 100 µM treated cells to vehicle-treated cells (*p* < 0.05, VIP > 1). Notably, doses below 10 µM showed an opposite effect, possibly indicating a biphasic effect of resveratrol due to a switch from anti-oxidant to pro-oxidant effects as dose levels increase. **Conclusions:** These findings suggest that resveratrol induces mitochondrial metabolic reprogramming in breast cancer cells in a dose-dependent manner. The biphasic response indicates a potential optimal dosage for therapeutic effectiveness. Further research is warranted to explore the mechanisms underlying these metabolic alterations and their implications for precision nutrition strategies in cancer treatment.

## 1. Introduction

Breast cancer is the most common cancer in women aside from nonmelanoma skin cancer, with approximately 2.3 million new cases and 685,000 deaths in 2020 [1]. Breast cancer risk factors include genetic mutations such as BRCA1 and BRCA2, prolonged exposure to estrogen due to early menarche, late menopause, and hormone replacement therapy, as well as lifestyle choices like alcohol consumption, physical inactivity, obesity, and consumption of a diet low in fruits and vegetables. Additionally, reproductive factors such as nulliparity, late age at first childbirth, and absence of breastfeeding, along with environmental exposures to ionizing radiation and endocrine-disrupting chemicals, contribute to increased risk [1,2]. Breast cancer is commonly categorized into three major subtypes: hormone receptor-positive (HR+), HER2-positive (HER2+), and triple-negative breast cancer (TNBC). HR+ breast cancer, which accounts for approximately 70% of cases, generally has a favorable prognosis with high responsiveness to endocrine therapy. HER2+ breast cancer makes up about 15–20% of cases and has historically had a poor prognosis, but outcomes have significantly improved with the advent of targeted HER2 therapies. TNBC, representing 10–15% of cases, is the most aggressive subtype and is associated with a poor prognosis, high rates of recurrence, and increased risk of metastasis, which is the primary cause of death in patients with TNBC [3,4]. The treatment of TNBC is challenging, particularly as it is commonly treated with high-dose chemotherapies, since targeted therapies are not available, and as a result, patients often see high rates of recurrence with a disease that is drug-resistant and prone to metastasis [5]. Although some subtypes of breast cancer have more favorable outcomes than others, the burden of breast cancer overall on the global population remains high, and new methods to treat or prevent breast cancer are greatly needed.

Polyphenols are natural compounds found in many fruits, vegetables, and other plant-based foods that are potent antioxidants and have been shown to possess many anticancer properties [6]. Several studies suggest that dietary polyphenols may have the potential to inhibit tumor growth and metastasis in breast cancer [7,8,9,10]. However, the effectiveness of polyphenols in TNBC is not well understood, particularly because these compounds are highly pleiotropic, often altering the activity of multiple targets in cancer cells [11]. Furthermore, polyphenols (and other phytochemicals) are generally well tolerated, have minimal side effects compared to traditional cancer therapies, and can protect against toxicities of many standard chemotherapies or even enhance their efficacy [12,13,14,15,16], making them attractive options for novel anticancer therapeutics.

One of the most well-studied polyphenols is resveratrol, a stilbene polyphenol [17]. Resveratrol is found predominantly in grapes, berries, and peanuts and has garnered significant attention due to its beneficial effects in cardiovascular disease, cancer, neurodegenerative disorders, and others. Known for its potent antioxidant and anti-inflammatory effects, resveratrol has been extensively studied for its role in cancer prevention and treatment [18]. Resveratrol has been shown to have mixed estrogenic/antiestrogenic activity, which has been shown to play a role in inhibiting estrogen-mediated cell proliferation in ER+ breast cancer cells. Resveratrol also inhibits cell proliferation, induces apoptosis, and suppresses metastasis in TNBC cells, indicating that there are anticancer activities of resveratrol that occur independently of estrogen receptor interactions [19,20]. Additionally, the capability of resveratrol and other polyphenols to enhance the efficacy of conventional chemotherapeutic agents makes these compounds compelling candidates for combination therapies in breast cancers as well as cancers of other types [21].

In addition to its effect on many cell signaling pathways, resveratrol has been shown to be a potent modulator of metabolism, mimicking features of caloric restriction and being beneficial for combating features of metabolic syndrome. Cancer cells exhibit altered metabolic processes, such as the Warburg effect, where they often rely heavily on glycolysis for energy production even in the presence of oxygen. Metabolic reprogramming events such as this and many others support rapid cell growth and survival during many stages of cancer initiation and progression. Specifically, resveratrol has been observed to inhibit glycolytic enzymes such as hexokinase and lactate dehydrogenase, reducing glycolytic flux and thereby limiting the energy supply to cancer cells [22]. Furthermore, resveratrol enhances mitochondrial biogenesis and oxidative phosphorylation, which increases the generation of reactive oxygen species (ROS), leading to oxidative stress and cancer cell death [23]. Moreover, resveratrol has been shown to inhibit fatty acid synthesis and promote lipolysis and fatty acid oxidation [24]. A previous targeted metabolomics study of 54 analytes was performed on resveratrol-treated breast cancer cells and found that resveratrol increased levels of amino acids, biogenic amines, and eicosanoids/oxylipins [25]. However, this was largely based on media metabolites, and approximately half of the metabolites were not detected in the cells while the remaining half largely did not produce significant intracellular changes. This, along with the relatively small panel of analytes measured, leaves our understanding of how resveratrol alters cellular metabolism in breast cancer very limited.

Because of the pleiotropic nature of resveratrol, it is difficult to ascertain which of these metabolic effects are the most prominent following the treatment of breast cancer cells with this compound. This information, when discovered, could lead to the development of new therapeutics or could inform of more effective ways to administer resveratrol, such as selecting patients with favorable genotypes or identifying combinations that would likely enhance resveratrol’s effect. This paper aims to use an untargeted metabolomics approach to simultaneously capture the totality of resveratrol’s effects on the metabolism of two commonly studied breast cancer cell lines: MCF-7 (HR+) and MDA-MB-231 (triple-negative). Through this approach, we seek to provide a comprehensive overview of resveratrol’s effect on breast cancer metabolism and to also identify the primary metabolic targets of this polyphenol. This information will help in better understanding how this compound exerts anticancer effects and how it can best be utilized either through diet or supplements to treat or prevent cancer, possibly in combination with other therapeutic agents.

## 2. Methods

### 2.1. Materials

Optima grade water and methanol containing 0.1% formic acid and fetal bovine serum (FBS) were purchased from Fisher Scientific (Waltham, MA, USA). Dubelcco’s Modified Eagle Medium (DMEM) with high glucose and phosphate-buffered saline (PBS) was purchased from Gibco (Grand Island, NY, USA). Trans-resveratrol was purchased from Cayman Chemical (Ann Arbor, MI, USA). MCF-7 and MDA-MB-231 cell lines were purchased from the American Type Culture Collection (ATCC) (Manassas, VA, USA).

### 2.2. Cell Culture

MCF-7 and MDA-MB-231 cells were cultured using DMEM supplemented with 10% FBS, 2 mM glutamine, 50 U/mL penicillin, and 50 μg/mL streptomycin. Cells were maintained in humidity-controlled CO_2_ incubators at 37 °C and 5% CO_2_.

### 2.3. Treatment of MCF-7 and MDA-MB-231 Cells with Resveratrol

The day before treatment, 1 × 10^6^ cells were plated into 6 well plates and were allowed to attach overnight. Resveratrol was dissolved in DMSO and added to culture wells at a concentration of 0, 1, 10, or 100 µM for 24 h for MCF-7 cells, and 0, 25, 50, or 100 µM for 24 h for MDA-MB-231 cells. Final DMSO concentration was 0.1% for all treatments (n = 3 per treatment group). To generate replicates for each group, separate wells were seeded from the same original cell line aliquot and treated separately.

### 2.4. Metabolite Extraction

Metabolites were extracted according to previously published methods [10,26,27,28,29]. Briefly, after 24 h, plates were placed on ice, treatment media was removed, and cells were washed with 1 mL of ice-cold PBS. After removing PBS, 200 μL of ice-cold homogenization solution (80% methanol, 20% water) was added to each well, and cells were detached with the use of a cell scraper. Blank tubes were prepared by adding 200 μL of homogenization solution to empty tubes. Protein concentration for lysates was measured by drying 20 μL aliquots of each sample by a SpeedVac, redissolving the protein in a 10% SDS solution, and analyzing by a bicinchoninic assay per manufacturer’s instructions (Thermo Fisher Scientific, San Jose, CA, USA). The remaining cell lysates were normalized to protein concentration by adding a proportional amount of additional homogenization solution. Samples were vortexed for 10 min at 5000 rpm on a multitube vortexer, and 10 μL was combined from each sample to make a quality control study pool (QCSP). All samples, blanks, and the QCSP were centrifuged at 16,000 rfc for 10 min at 4 °C. Supernatants were transferred to autosampler vials, and 5 μL was injected for analysis by ultra-high-performance liquid chromatography–high resolution mass spectrometry (UHPLC-HRMS).

### 2.5. UHPLC-HRMS Data Acquisition and Multivariate Statistics

Data were acquired using a Vanquish UHPLC system coupled to a Q Exactive™ HF-X Hybrid Quadrupole–Orbitrap Mass Spectrometer (Thermo Fisher Scientific, San Jose, CA, USA) according to previous methods [26,27,28,30,31]. An HSS T3 C18 column (2.1 × 100 mm, 1.7 µm, Waters Corporation, Milford, MA, USA) was used for metabolite separation. Mobile phases consisted of (A) water with 0.1% formic acid and (B) methanol with 0.1% formic acid. The solvent gradient was as follows: hold at 1% B for 1 min, increase to 99% B over 15 min, hold at 99% B for 4 min. The flow rate throughout the UHPLC analysis was 400 µL/min. Data-dependent acquisition of the untargeted data was performed from 70 to 1050 *m*/*z* in positive mode. Additional mass spectrometer settings were as follows: resolution = 120,000, microscans = 1, AGC target = 3 × 10^6^, sheath gas flow rate = 55 L/min, auxiliary gas flow rate = 15 L/min, sweep gas flow rate = 2 L/min, spray voltage = 3.50 kV, capillary temperature = 320 °C, and auxiliary gas heater temperature = 400 °C. Peak picking, alignment, and normalization were performed using Progenesis QI using the “normalize to all” function with a QCSP sample selected as the reference (version 2.1, Waters Corporation, Milford, MA, USA) [32]. MCF-7 and MDA-MB-231 experiments were processed separately. Background signals were removed by filtering out peaks with a higher average abundance in the blanks as compared to the QCSP. Normalized, preprocessed data were analyzed by Principal Component Analysis (PCA) and Orthogonal Partial Least Squares–Discriminant Analysis (OPLS-DA) using SIMCA 16 (Sartorius Stedim Data Analytics AB, Umeå, Sweden).

### 2.6. Pathway Analysis

A peak-intensity table was generated using all of the normalized, preprocessed peaks and was then input into the “Functional Analysis” module of MetaboAnalyst 5.0 using a mass tolerance of 5 ppm. Pathway significance was calculated using the default 10% of peaks by *p*-value. The Homo sapiens (human) [MFN] option was selected as the pathway library for the analysis.

### 2.7. Metabolite Identification

Metabolites were identified and annotated in Progenesis QI by matching to in-house metabolite libraries. Peaks were matched to metabolites by retention time (RT, ±0.5 min), exact mass (MS, <5 ppm), and/or fragmentation pattern (MS/MS, similarity score > 30). An ontology system is given to denote the evidence basis for a given metabolite match. OL1 refers to an in-house metabolite match by RT, MS, and MS/MS; OL2a refers to an in-house match by RT and MS only; OL2b refers to an in-house match by MS and MS/MS only. Additionally, peaks were matched to public databases (NIST and HMDB). The NIST database contains *m*/*z* and MS/MS data, whereas HMDB contains only *m*/*z*. PDA refers to a public database match by MS and MS/MS (NIST only); PDC refers to a public database match by MS with high isotopic similarity (>90%); PDD refers to a public database match by MS with low isotopic similarity (<90%). The following adducts were considered for peak matching: M+H, M+Na, M+H-H_2_O; M+e; M+K; 2M+Na; M+CH_3_OH+H; M+H+Na; M+H-NH_3_; M+2Na; M+H-2H_2_O; 2M+H; M+2H; M+2Na-H; M+2H+Na; M+2Na+H; and M+NH_4_.

### 2.8. Univariate Statistics

Peaks that matched to a metabolite by an OL1, OL2a, or OL2b ontology level were input into MetaboAnalyst’s “Statistical Analysis” module as a peak-intensity table using the normalized, preprocessed data. Metabolite heatmaps, ANOVA plots, and box-and-whisker plots were generated in MetaboAnalyst. Pearson correlation coefficients and *p*-values between metabolite peak areas and resveratrol concentrations were calculated using MetaboAnalyst with the resveratrol dose as a continuous variable.

## 3. Results

**Figure 1:**
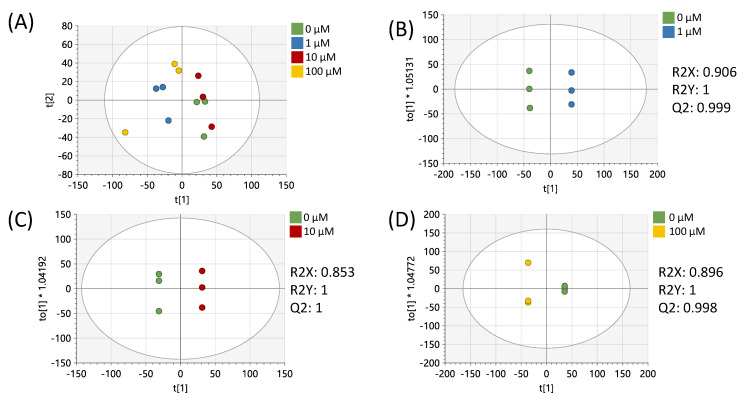
Multivariate analysis using all peaks from MCF-7 samples. (**A**) PCA plot of all treatments. (**B**) OPLS-DA plot of cells treated with 0 or 1 µM of resveratrol. (**C**) OPLS-DA plot of cells treated with 0 or 10 µM of resveratrol. (**D**) OPLS-DA plot of cells treated with 0 or 100 µM of resveratrol. All multivariate plots were scaled to unit variance. Green = 0 μM, blue = 1 μM, red = 10 μM, yellow = 100 μM.

Data preprocessing and filtering of samples derived from MCF-7 cells resulted in 4751 peaks in the metabolomics dataset. Of these peaks, 312 matched to the in-house standards library and 3459 matched to public databases. PCA of study samples and QCSPs using all peaks in the dataset showed tight clustering of QCSPs in the center of the study samples (Supplementary Figure S1). Analysis of study samples by PCA in Figure 1A using all peaks showed that the 1 µM and 100 µM treatments produced metabolic profiles most distinct from the vehicle control, in contrast to the 10 µM treated samples which appeared more similar to the vehicle. Pairwise OPLS-DA models showed that each treatment compared to the vehicle showed significant differences in metabotypes (R2Y = 1) with high cross-validation scores (Q2 > 0.9) (Figure 1B–D). *p*-values (calculated by Students’ *t*-test), fold changes, and variable importance to projection scores (VIP, derived from OPLS-DA plots) were calculated between the vehicle control and each resveratrol treatment for each peak (Supplementary Table S1). These results showed 1153, 394, and 904 peaks had a *p* < 0.05 between vehicle-treated cells and 1, 10, and 100 µM resveratrol-treated cells, respectively.

**Figure 2:**
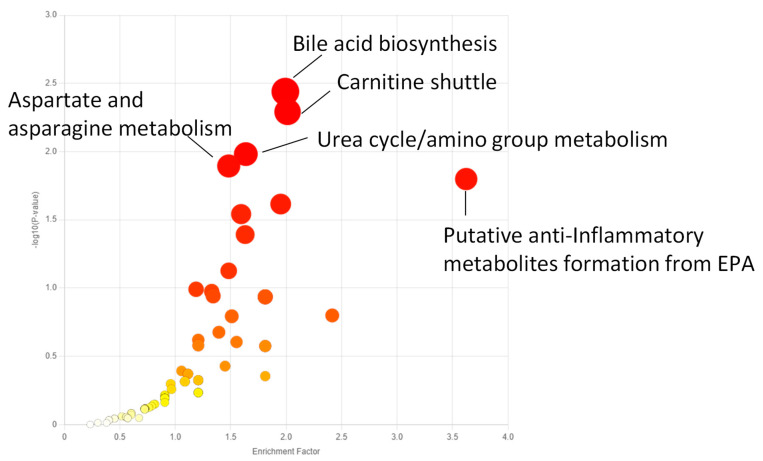
Enriched metabolic pathways across all treatment groups in MCF-7 samples using the Functional Analysis module in MetaboAnalyst. All peaks for all four treatment groups were uploaded as a peak-intensity table in MetaboAnalyst, and ANOVA *p*-values were used to determine peaks significantly altered by treatment. The top five pathways by *p*-value are annotated. A full list of significantly enriched pathways is listed in Table 1. Darker red indicates a larger −log_10_ (*p*-value).

**Figure 3:**
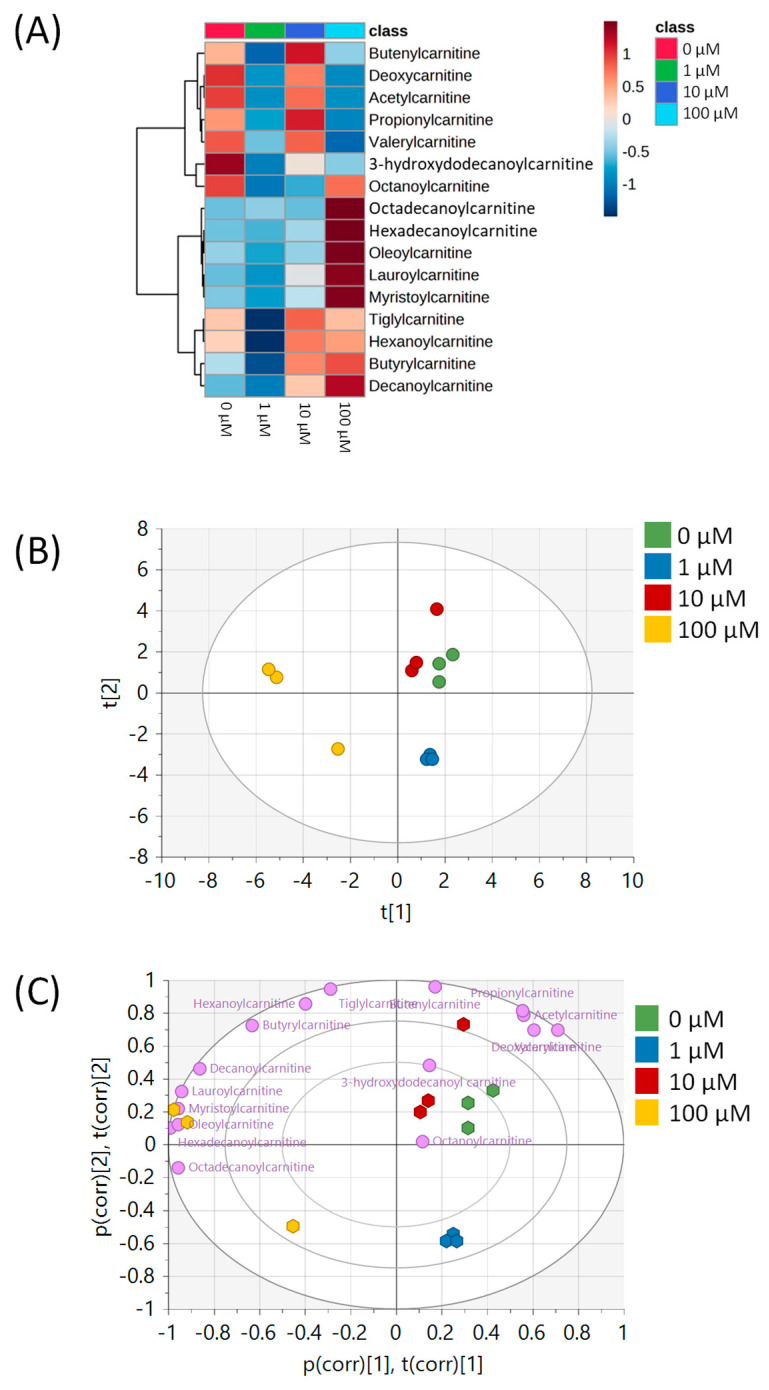
Analysis of carnitine/acylcarnitine metabolites in MCF-7 samples. (**A**) Heatmap of 16 carnitine/acylcarnitine metabolites across all four treatment groups. Data are auto-scaled (mean-centered and divided by standard deviation) for each metabolite. (**B**) PCA plot of all four treatment groups using the 16 carnitine/acylcarnitine metabolites. Green = 0 μM, blue = 1 μM, red = 10 μM, yellow = 100 μM. (**C**) Biplot showing the loadings of each carnitine/acylcarnitine metabolite on top of the PCA from Figure 3B. Individual metabolites are indicated in purple. All multivariate plots were scaled to unit variance. Axis labels p(corr) [1],t(corr) [1] and p(corr) [2],t(corr) [2] refer to the correlation-scaled loadings (p) and scores (t) of the first and second principal components, respectively.

**Figure 4:**
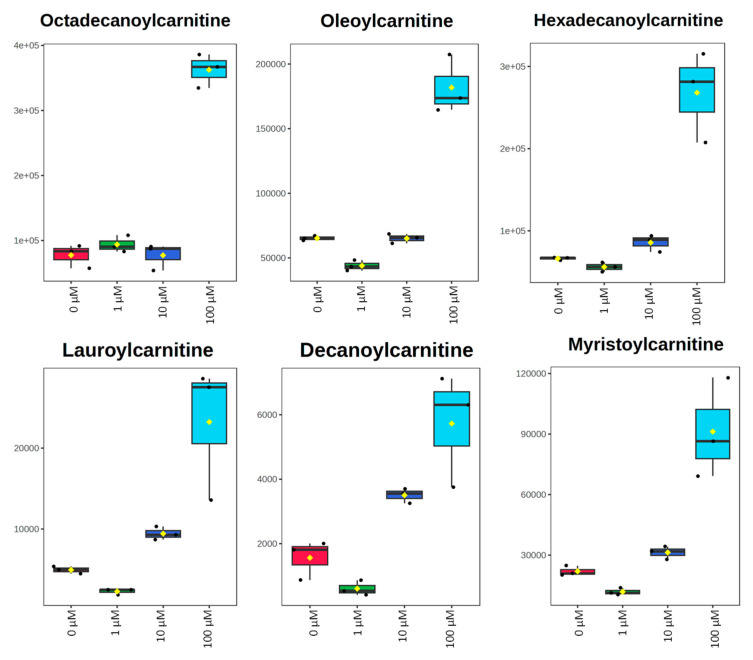
Box plots of peak-abundance values from long-chain acylcarnitines in MCF-7 samples across all four treatment groups. Red = 0 μM, green = 1 μM, dark blue = 10 μM, light blue = 100 μM.

All metabolomic features (4751 peaks) and peak intensities for all four treatment groups were input into MetaboAnalyst for pathway analysis using the Mummichog algorithm. Using ANOVA *p*-values for all peaks, eight pathways were found to be significant (*p* < 0.05) across the treatment groups, as shown in Figure 2 as well as Table 1. These significant pathways represented steroid, fatty acid, amino acid, and nucleotide metabolic pathways. Since Mummichog primarily only uses mass to annotate peaks to compounds, we cross-referenced peak matches to our in-house reference standard library to determine if significant perturbations in these pathways could be validated. Our data showed 16 carnitine/acylcarnitine metabolites matched to the in-house library that showed significant differences between treatment groups (Figure 3A). Overall, these metabolites showed that short-chain (SC) and medium-chain (MC) acylcarnitines decreased in abundance, while long-chain (LC) acylcarnitines increased in abundance as resveratrol concentration increased. PCA of only these 16 carnitine/acylcarnitine metabolites (Figure 3B) showed a similar trend between treatments, as seen with all peaks (Figure 1A), although a smaller within-group variation was seen in the treatment groups. Biplot analysis showed that an elevated LC acylcarnitine signature was associated with the 100 µM treatment group (Figure 3C). A closer look at LC acylcarnitines showed strong increases in the levels of these metabolites in the 100 µM dose group (Figure 4, Table 2). Fold-change increases in these metabolites in the 100 µM treated group as compared to the vehicle group ranged from ~2–5, with significant *p*-values (*p* < 0.05) and variable importance in projection (VIP) scores (VIP > 1). The VIP score is a measure of how important a variable is for distinguishing between groups in the OPLS-DA model, with higher scores indicating a stronger contribution to differentiation. Interestingly, some of these LC acylcarnitine metabolites showed a biphasic response across all three resveratrol dose levels, where they decreased in the 1 µM treated group, returned to near baseline in the 10 µM treated group, and strongly increased in the 100 µM treated group. This observation was seen for many other metabolites in addition to the carnitines/acylcarnitines (Supplementary Table S1). This observation is in agreement with many other studies showing a hormesis-like biphasic response of resveratrol, where lower doses produce an antioxidant effect and high doses produce a pro-oxidant effect [33,34,35,36,37]. The 10 µM treated samples appear to be close to this inflection point, which may be the reason why the 10 µM treated samples were more similar to the vehicle-treated samples in the PCA plots.

**Figure 5:**
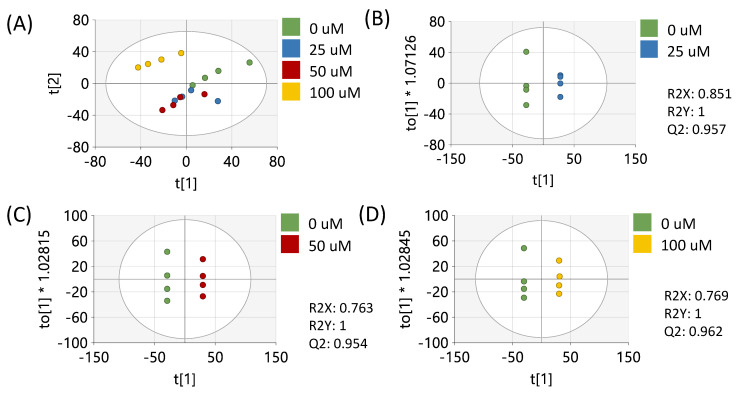
Multivariate analysis using all peaks from MDA-MB-231 samples. (**A**) PCA plot of all treatments. (**B**) OPLS-DA plot of cells treated with 0 or 25 µM of resveratrol. (**C**) OPLS-DA plot of cells treated with 0 or 50 µM of resveratrol. (**D**) OPLS-DA plot of cells treated with 0 or 100 µM of resveratrol. All multivariate plots were scaled to unit variance. Green = 0 μM, blue = 25 μM, red = 50 μM, yellow = 100 μM.

To further investigate the metabolic effects of resveratrol and to determine if the metabolic effects are similar in other breast cancer types, we treated MDA-MB-231 cells with 0, 25, 50, or 100 µM of resveratrol and performed untargeted metabolomics analysis on these samples. Because we observed a biphasic effect in the 1, 10, and 100 µM doses in the MCF-7 samples, these new concentrations were chosen so that we could further investigate the monophasic response of metabolites with higher doses of resveratrol. Following data preprocessing, 3156 peaks remained in the dataset. Of these, 248 peaks were matched to the in-house library, and 2010 were matched to public databases (Supplementary Table S2). PCA of all study samples showed separation between the vehicle and each treatment group, with the top dose showing the greatest separation from the vehicle control (Figure 5A). Pairwise OPLS-DA plots showed significant differentiation (R2Y = 1) between the vehicle and each treatment with high cross-validation scores (Q2 > 0.9) (Figure 5B–D). Fold changes, *p*-values, and VIP scores for each of the 3156 peaks were calculated between the vehicle and each dose and are listed in Supplementary Table S2. These results showed that 746, 812, and 942 peaks had a *p* < 0.05 between vehicle-treated cells and 25, 50, and 100 μM resveratrol-treated cells, respectively.

**Figure 6:**
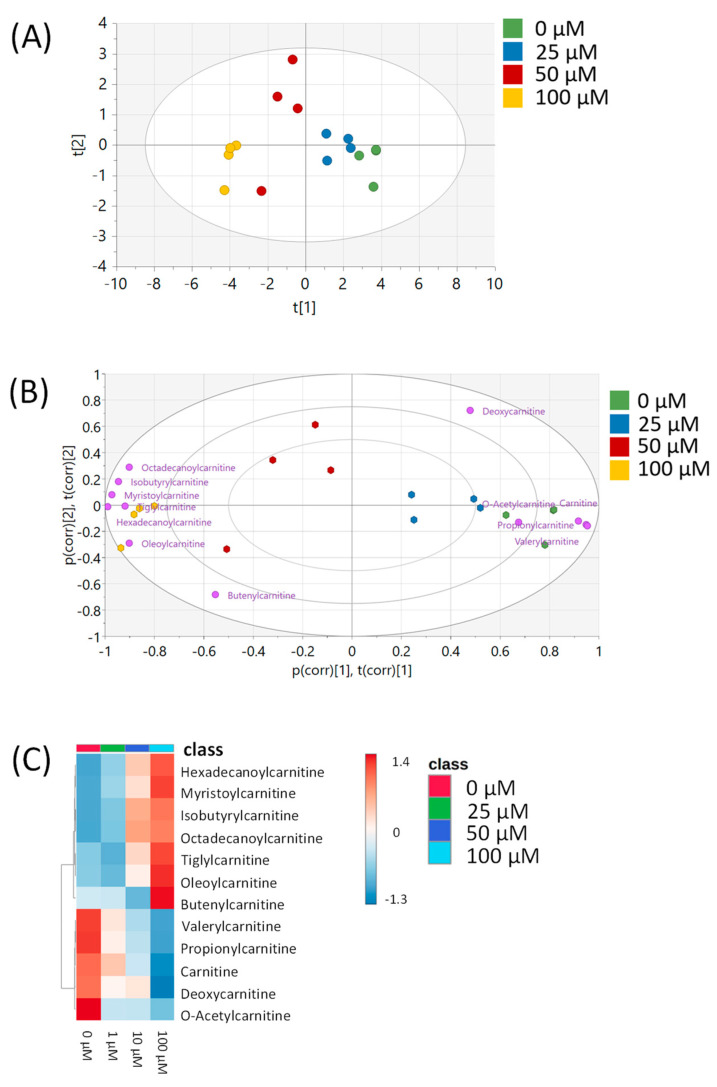
Analysis of carnitine/acylcarnitine metabolites in MDA-MB-231 samples. (**A**) PCA plot of all four treatment groups using the 12 carnitine/acylcarnitine metabolites. Green = 0 μM, blue = 25 μM, red = 50 μM, yellow = 100 μM. (**B**) Biplot showing the loadings of each carnitine/acylcarnitine metabolite on top of the PCA from Figure 6A. Individual metabolites are indicated in purple. Axis labels p(corr) [1],t(corr) [1] and p(corr) [2],t(corr) [2] refer to the correlation-scaled loadings (p) and scores (t) of the first and second principal components, respectively. (**C**) Heatmap of 12 carnitine/acylcarnitine metabolites across all four treatment groups. All multivariate plots were scaled to unit variance. Data are auto-scaled (mean-centered and divided by standard deviation) for each metabolite.

**Figure 7:**
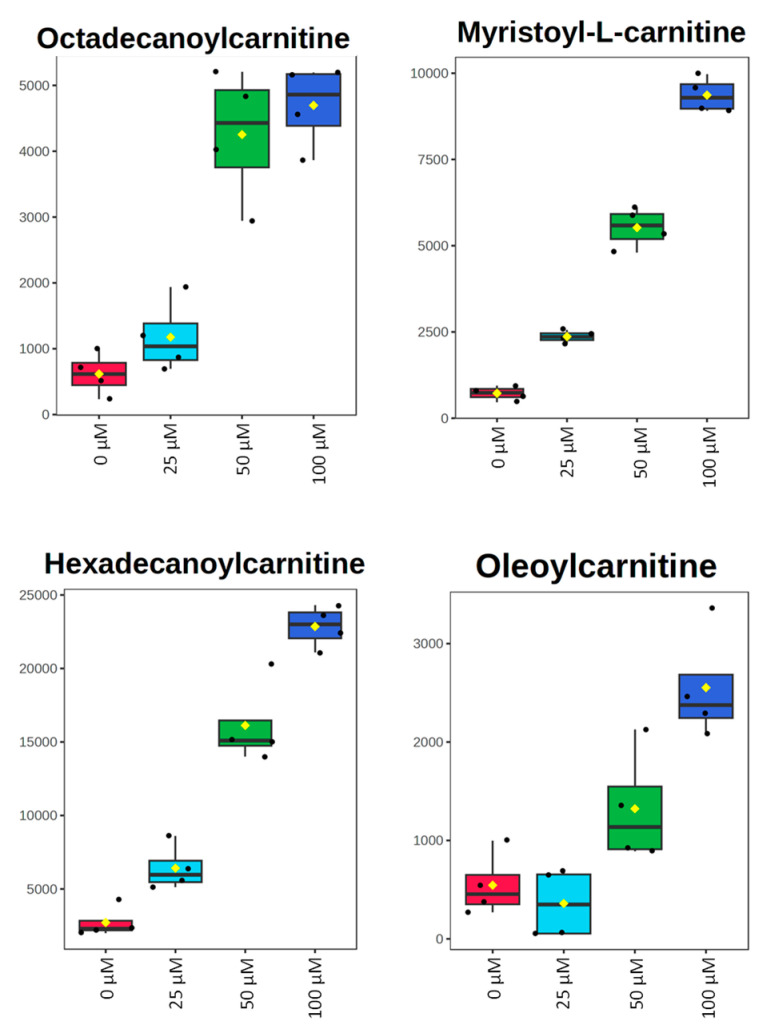
Box plots of peak-abundance values from long-chain acylcarnitines in MDA-MB-231 samples across all four treatment groups. Red = 0 μM, light blue = 25 μM, green = 50 μM, dark blue = 100 μM.

Twelve carnitine/acylcarnitine metabolites matched to the in-house library in this dataset. PCA of all samples using only carnitine/acylcarnitine metabolites showed greater resolution between dose levels, as compared to the PCA plot of all peaks in the dataset (Figure 6A). Overall patterns of these metabolites, as investigated by heatmap and biplot analyses, showed an increase in LC acylcarnitines and a decrease in SC and MC acylcarnitines at higher doses of resveratrol (Figure 6B,C). The LC acylcarnitines showed larger magnitudes of fold changes in MDA-MB-231 cells, as compared to the MCF-7 cells, with values ranging from ~5 to 13-fold increases in the 100 µM dose group, as compared to the vehicle control, while also having significant *p*-values (*p* < 0.05) and VIP scores (VIP > 1) (Figure 7 and Table 3).

## 4. Discussion

The present study investigates the metabolic effects of resveratrol treatment in MCF-7 and MDA-MB-231 cell lines using concentrations across a dose range of 0 to 100 µM. Certain breast cancers, such as triple-negative breast cancer (TNBC), have poor prognoses and few targeted therapies available. Targeting metabolism may offer a more effective approach to treating these aggressive breast cancers. Resveratrol, a natural polyphenol, has been commonly studied for its potential against cancers, including breast cancers. While resveratrol is well recognized to have pleiotropic effects, it has been shown to target aspects of metabolic processes in breast cancers. A study using mitochondria-targeted delivery of resveratrol via triphenylphosphonium (TTP) conjugation in murine 4T1 and human MDA-MB-231 breast cancer cell lines revealed significant alterations in the pentose phosphate pathway and upregulation of glycerophospholipids. This study similarly utilized an untargeted metabolic approach for metabolite analysis but focused on the mitochondria for treatment, rather than targeting the cell as a whole [38]. In a separate study, resveratrol was found to notably disrupt glucose metabolism by inhibiting the activity of the enzyme 6-phosphofructo-1-kinase (PFK) in MCF-7 breast cancer cells. The analysis utilized an MTT assay to assess cellular viability and did not examine the individual metabolites affected [39]. Given the various ways in which resveratrol can alter metabolism, determining the mechanisms behind these changes could lead to alternative breast cancer treatments, whether nutritional or pharmacological.

The metabolomic approach used in this study showed that treatment of breast cancer cells with resveratrol significantly affected the metabolome. Particularly, multivariate analysis of metabolites in MCF-7 and MDA-MB-231 cells highlights that low, medium, or high doses of resveratrol all had significant effects on the metabolome of MCF-7 and MDA-MB-231 cells as compared to vehicle controls. Pathways most significantly impacted following resveratrol treatment in MCF-7 cells included bile acid biosynthesis, carnitine shuttle, urea cycle/amino group metabolism, aspartate and asparagine metabolism, and putative anti-inflammatory metabolites formed from eicosapentaenoic acid (EPA). Further analysis of individual metabolites in these pathways for both MCF-7 and MDA-MB-231 cells found significant differences in various acylcarnitine metabolites. In both cell lines, as the concentration of resveratrol treatment increases, long-chain acylcarnitines such as octadecanoylcarnitine, myristoylcarnitine, hexadecanoylcarnitine, and oleoylcarnitine increase, and short/medium chain acylcarnitines such as butenylcarnitine, acetylcarnitine, and propionylcarnitine decrease. Interestingly, a biphasic effect on the metabolome was seen with resveratrol concentrations in the range of 1–10 µM. For instance, compared to the vehicle control, acylcarnitines in MCF-7 cells decreased in the 1 µM dose, returned to baseline at the 10 µM dose, and then increased significantly in the 100 µM dose. This trend merits attention for future studies, as determining the optimal amount of dosage concentration is essential for future therapeutic development.

Investigation of acylcarnitines is especially relevant, as these metabolites are directly related to mitochondrial function, fatty acid metabolism, and overall energy production within cells [40]. Understanding how resveratrol influences acylcarnitine levels can reveal its broader impact on cellular energy dynamics and cancer cell adaptability. Indeed, the carnitine system has been recognized as a major factor in cancer cell metabolic plasticity, allowing cancer cells to adjust bioenergetic demands in response to internal and external stressors [41]. Derived from fatty acids, acylcarnitines are converted forms of fatty acids with increased polarity to translocate into the mitochondrial matrix to undergo beta-oxidation. As acylcarnitines undergo beta oxidation and ATP is synthesized, the acyl chains become shorter as the molecule goes through an increased number of oxidation cycles [40,42]. As determined by our study, increased dosages of resveratrol lead to a trend of elevated levels of long-chain acylcarnitines. This signifies that there is a likely disruption in mitochondrial function as an accumulation of unprocessed fatty acids, in the form of LC acylcarnitines, which occurs because of incomplete beta-oxidation. Long-chain acylcarnitines are often characteristic of kidney disease, fatty liver disease, insulin resistance, and more, indicating the importance of the carnitine system in overall metabolic health [43,44,45,46]. The connection with resveratrol and mitochondrial function/lipid metabolism is supported through numerous studies. In a study of human fibroblasts, it was shown that resveratrol can modulate fatty acid oxidation activity [47]. Resveratrol has also been shown to activate fatty acid mobilization and inhibit fatty acid synthesis in an experiment with healthy mice [24] and is known to increase mitochondrial biogenesis through activation of SIRT1 [48]. Particularly aligned with our focus on breast cancer treatment, Khan et al. found that treatment of resveratrol downregulated fatty acid synthase and ultimately led to cell death in HER2-positive breast cancer [49]. Interestingly, we have also shown that changes in acylcarnitine profiles in breast cancer correlate with acquired drug resistance to chemotherapies [30]. Overall, targeting the carnitine system may be a major mechanism behind resveratrol’s anticancer effects and may also provide a rationale for combining resveratrol with other agents that induce energetic stress. We have observed that other dietary phytochemicals such as curcumin, genistein, and tannic acid also affect acylcarnitine profiles in a similar manner, which may indicate the potential for combining these agents for improved therapeutic effects [10]. While this pattern of acylcarnitines has not been studied in detail in the context of cancer, it has been shown that, in diabetes, disruptions in the acylcarnitine pool are well-established indicators of mitochondrial dysfunction and the decoupling of fatty acid oxidation (FAO) from oxidative phosphorylation [50]. Elevated acylcarnitine levels arise when FAO outpaces the TCA cycle, leading to increased lipolysis and incomplete mitochondrial substrate oxidation. When substrate breakdown exceeds ATP demand, heightened reducing pressure from NADH and FADH2 on the electron transport chain promotes reactive oxygen species (ROS) production (H_2_O_2_, •O_2_) [51]. Excessive mitochondrial ROS and acyl accumulation can trigger the opening of the mitochondrial permeability transition pore (PTP), ultimately resulting in cell death [50,52,53,54,55]. Our findings indicate that a similar process may occur in cancer cells treated with these polyphenols, which may explain the pro-oxidant effects of these molecules at higher doses and in tumor environments [56]. Given that resveratrol has been shown to be selectively toxic to cancer cells through mitochondrial and endoplasmic reticulum-targeting effects [57], these pathways may be metabolic vulnerabilities of cancer cells that can be exploited to increase the efficacy and selectivity of breast cancer treatments.

Acylcarnitines have emerged as key biomarkers in precision medicine to potentially indicate disease severity and treatment response [58]. While the current manuscript investigates the link between acylcarnitines and resveratrol in cultured breast cancer cells, the results may have important implications for individuals with breast cancer. Our findings suggest that individuals with breast tumors that show greater defects in fatty acid and acylcarnitine metabolism may respond more effectively to resveratrol treatment, as our study showed that this pathway was highly targeted in our cell models. Such an approach not only opens new avenues for targeted breast cancer therapies but also emphasizes the importance of individual metabolic profiles in developing tailored treatment strategies. Assessing an individual’s tumor acylcarnitine profile could identify candidates who might benefit most from resveratrol treatment. The observed biphasic response in acylcarnitine levels in breast cancer cells following resveratrol treatment also suggests that doses could be tailored to optimize treatment efficacy based on these acylcarnitine profiles. By integrating patient-specific metabolic profiles into treatment plans, we may enhance the efficacy of resveratrol and improve outcomes for those facing challenging breast cancer prognoses. However, clinical measurements of acylcarnitines are primarily performed using serum/plasma/urine for screening of genetic disorders in newborns, indicating that new clinical acylcarnitine tests for tumors may need to be developed for this purpose [59]. This also may improve our ability to make dietary recommendations for cancer therapy, as diets high in resveratrol may be beneficial in individuals with tumors showing greater mitochondrial dysfunction. The Mediterranean diet is a prime example of this kind of diet, as it emphasizes resveratrol-rich options like red wine, grapes, and berries [60]. Importantly, we have shown in previous studies that other dietary phytochemicals also target acylcarnitine profiles in a manner similar to resveratrol [10], indicating that multiple dietary bioactive substances in the diet may act synergistically to improve targeting of this pathway. More work is needed to validate the role of acylcarnitines in patient tumors in order to translate our findings.

## 5. Conclusions

The results of our study show that resveratrol significantly alters the metabolome of MCF-7 and MDA-MB-231 cells with particularly profound effects on the carnitine system. The carnitine system has been widely recognized as a central regulator and determinant of metabolic adaptability of cancer cells [41]. Thus, this finding not only sheds light on the mechanisms of resveratrol’s action but also suggests potential pathways for developing therapeutic strategies aimed at exploiting the metabolic vulnerabilities of breast cancer cells. This is the first study that has revealed that acylcarnitines serve as a central metabolic target of resveratrol in both TNBC and HR+ breast cancer cells in a dose-dependent manner, providing more insights towards the primary mechanism of action of this pleiotropic molecule. These findings enhance our understanding of how to design therapeutics that mimic the action of resveratrol and the metabolic perturbations that are associated with its treatment. While our study focused on carnitine/acylcarnitine metabolites, it should be noted that many other metabolic changes occurred following resveratrol treatment (as seen in the supplementary tables) which could contribute to the anticancer effects of this molecule. Future studies should investigate if these effects can also be seen in vivo and if other agents that target the carnitine system work synergistically with resveratrol and other related polyphenols.

## Figures and Tables

**Table 1:** Significantly perturbed metabolic pathways (*p* < 0.05) across all treatment groups in MCF-7 cells, as calculated by the Mummichog algorithm for pathway analysis in MetaboAnalyst.

Pathway Name	Pathway Total ^a^	Hits.total ^b^	Hits.sig ^c^	FET ^d^
Bile acid biosynthesis	82	35	27	0.0036
Carnitine shuttle	72	18	14	0.0051
Urea cycle/amino group metabolism	85	25	14	0.0104
Aspartate and asparagine metabolism	114	45	20	0.0128
Putative anti-inflammatory metabolite formation from EPA	27	2	2	0.0159
Glutathione metabolism	19	9	6	0.0243
Purine metabolism	80	19	12	0.0288
Lysine metabolism	52	16	7	0.0406

^a^ Pathway total indicates the total number of metabolites for a specific pathway in the database. ^b^ Hits.total indicates the number of experimental signals that matched (*m*/*z* error  <  5 ppm) with metabolites included in the pathway. ^c^ Hits.sig indicates the number of matched metabolites with a significant ANOVA *p*-value across treatment groups. ^d^ FET is the right-tail *p*-value determined by the Fisher Exact Test for pathway enrichment.

**Table 2:** Changes in carnitine metabolites in MCF-7 cells treated with varying doses of resveratrol.

		Fold Change			*p*-Value			VIP		
Metabolite Name	Ontology Level	1 µM/ 0 µM	10 µM/ 0 µM	100 µM/ 0 µM	0 µM vs. 1 µM	0 µM vs. 10 µM	0 µM vs. 100 µM	0 µM vs. 1 µM	0 µM vs. 10 µM	0 µM vs. 100 µM
Lauroylcarnitine	OL2b	0.46	1.91	4.70	1.3 × 10^−3^	1.2 × 10^−3^	0.020	1.33	1.58	1.32
Octadecanoylcarnitine	OL2b	1.21	1.00	4.67	0.27	0.99	9.8 × 10^−5^	0.90	0.77	1.43
Myristoylcarnitine	OL2b	0.54	1.42	4.15	3.8 × 10^−3^	0.015	8.5 × 10^−3^	1.31	1.46	1.35
Hexadecanoylcarnitine	OL2b	0.84	1.29	4.04	0.040	0.031	3.2 × 10^−3^	1.14	1.43	1.39
Decanoylcarnitine	OL2b	0.39	2.24	3.67	0.063	6.5 × 10^−3^	0.018	1.16	1.53	1.33
Oleoylcarnitine	OL2b	0.67	1.00	2.79	1.2 × 10^−3^	0.95	8.6 × 10^−4^	1.33	0.77	1.41
Butyrylcarnitine	OL2a	0.47	1.47	1.60	2.1 × 10^−3^	3.4 × 10^−3^	0.29	1.32	1.56	0.99
Hexanoylcarnitine	OL2a	0.33	1.19	1.12	0.012	0.35	0.76	1.27	1.01	0.76
Tiglylcarnitine	OL1	0.38	1.18	1.01	1.3 × 10^−3^	0.25	0.96	1.33	1.10	0.73
Octanoylcarnitine	OL2a	0.43	0.53	0.94	0.61	0.68	0.97	0.80	0.81	0.75
Butenylcarnitine	OL2a	0.36	1.31	0.67	2.0 × 10^−3^	0.30	0.16	1.32	1.04	1.10
Acetylcarnitine	OL1	0.44	1.22	0.57	3.9 × 10^−3^	0.30	0.035	1.31	1.05	1.28
Deoxycarnitine	OL1	0.57	0.92	0.56	0.010	0.57	0.015	1.27	0.87	1.33
3-OH-dodecanoylcarnitine	OL2a	0.21	0.54	0.39	0.17	0.45	0.31	1.03	0.65	0.96
Propionylcarnitine	OL1	0.38	1.26	0.29	1.7 × 10^−3^	0.30	2.0 × 10^−3^	1.32	1.04	1.40
Valerylcarnitine	OL1	0.48	0.98	0.24	1.4 × 10^−3^	0.87	8.1 × 10^−4^	1.33	0.77	1.42

**Table 3:** Changes in carnitine metabolites in MDA-MB-231 cells treated with varying doses of resveratrol.

		Fold Change			*p*-Value			VIP		
Metabolite Name	Ontology Level	25 µM/ 0 µM	50 µM/ 0 µM	100 µM/ 0 µM	0 µM vs. 25 µM	0 µM vs. 50 µM	0 µM vs. 100 µM	0 µM vs. 25 µM	0 µM vs. 50 µM	0 µM vs. 100 µM
Octadecanoylcarnitine	OL1	1.91	6.89	7.61	0.13	4.6 × 10^−4^	2.5 × 10^−5^	0.98	1.43	1.42
Oleoylcarnitine	OL1	0.61	2.43	4.68	0.43	0.057	7.9 × 10^−4^	0.87	1.18	1.37
Hexadecanoylcarnitine	OL1	2.36	5.93	8.40	7.4 × 10^−3^	1.2 × 10^−4^	4.6 × 10^−7^	1.37	1.47	1.45
Myristoylcarnitine	OL1	3.27	7.65	12.96	1.7 × 10^−5^	4.5 × 10^−6^	6.7 × 10^−8^	1.52	1.49	1.45
Butenylcarnitine	OL2a	1.00	0.91	1.27	0.96	0.45	5.9 × 10^−3^	0.64	0.89	1.27
Valerylcarnitine	OL1	0.69	0.49	0.33	3.5 × 10^−4^	3.9 × 10^−6^	7.4 × 10^−7^	1.48	1.50	1.45
Tiglylcarnitine	OL1	0.91	1.37	1.71	0.15	7.2 × 10^−5^	1.6 × 10^−4^	1.06	1.47	1.41
Isobutyrylcarnitine	OL1	1.21	2.20	2.45	1.2 × 10^−3^	2.2 × 10^−7^	2.8 × 10^−7^	1.45	1.51	1.45
Propionylcarnitine	OL1	0.67	0.52	0.35	1.0 × 10^−5^	8.1 × 10^−8^	2.8 × 10^−9^	1.52	1.51	1.45
O-Acetylcarnitine	OL1	0.72	0.72	0.66	0.015	0.011	0.017	1.28	1.30	1.19
Carnitine	OL1	0.87	0.71	0.52	0.27	8.6 × 10^−3^	3.1 × 10^−4^	0.96	1.32	1.39
Deoxycarnitine	OL1	0.90	0.91	0.75	0.11	0.66	2.1 × 10^−2^	1.06	0.58	1.16

## Data Availability

All preprocessed data are available in the Supplementary Materials. Raw data are available upon request.

## Supplementary Materials

The following supporting information can be downloaded at: https://www.mdpi.com/article/10.3390/metabo15040250/s1. Figure S1: PCA plots of study samples and quality control study pools (QCSP) using all peaks from (A) MCF-7 and (B) MDA-MB-231 cells. All multivariate plots were scaled to unit variance; Table S1: Peak information and statistics for MCF7 samples; Table S2: Peak information and statistics for MDA-MB-231 samples.
