# Supplementary material for: Untargeted Metabolomics Reveals Acylcarnitines as Major Metabolic Targets of Resveratrol in Breast Cancer Cells
Source: Metabolites. 2025 Apr 5;15(4):250. doi: 10.3390/metabo15040250 (PMC12029535; doi:10.3390/metabo15040250)
Supplement: Supplementary file 1 [file metabolites-15-00250-s001.zip › Supplementary files/Supplementary Figure S1.pptx]

## Slide 1
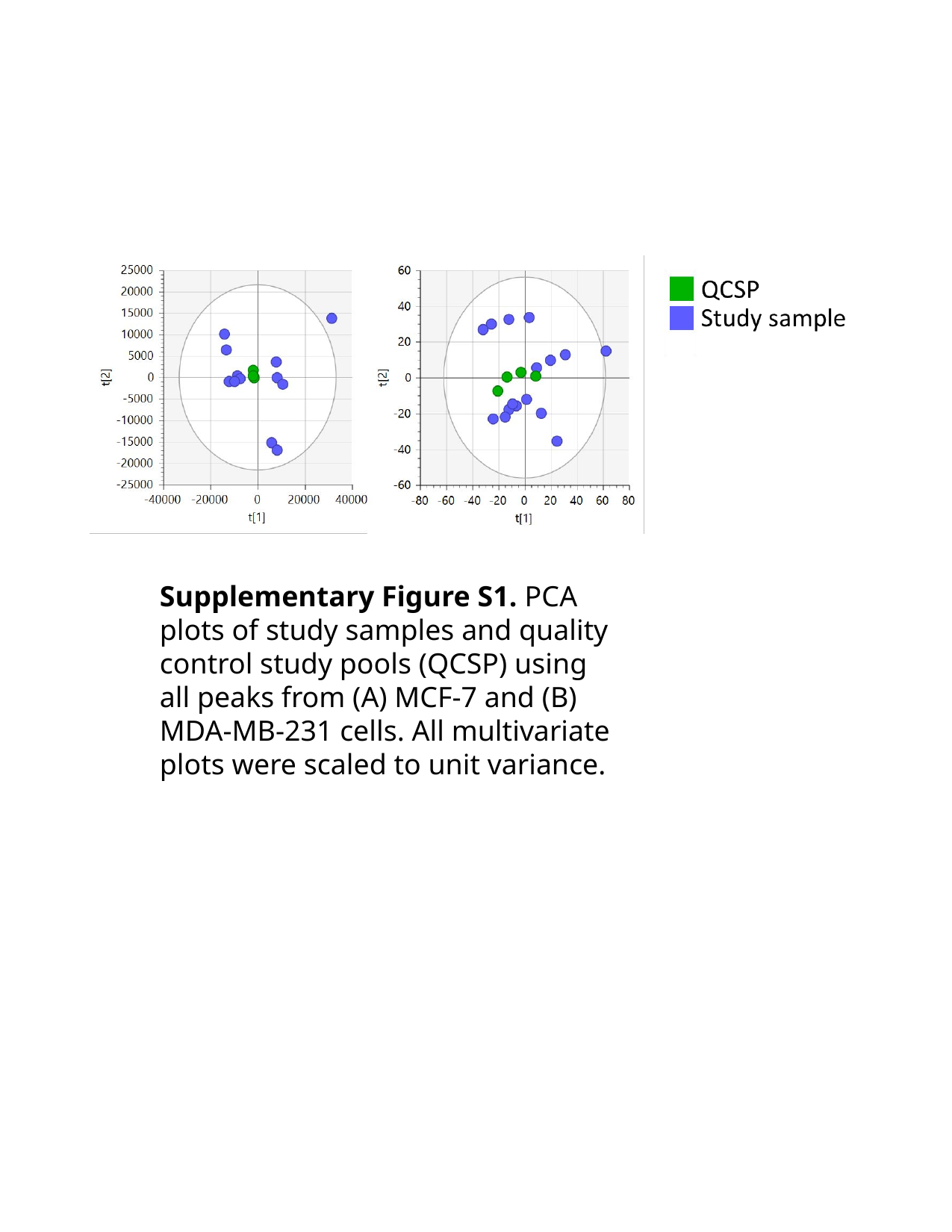

Supplementary Figure S1. PCA plots of study samples and quality control study pools (QCSP) using all peaks from (A) MCF-7 and (B) MDA-MB-231 cells. All multivariate plots were scaled to unit variance.
